# Exercise training in the aerobic/anaerobic metabolic transition prevents glucose intolerance in alloxan-treated rats

**DOI:** 10.1186/1472-6823-8-11

**Published:** 2008-10-02

**Authors:** Clécia Soares de Alencar Mota, Carla Ribeiro, Gustavo Gomes de Araújo, Michel Barbosa de Araújo, Fúlvia de Barros Manchado-Gobatto, Fabrício Azevedo Voltarelli, Camila Aparecida Machado de Oliveira, Eliete Luciano, Maria Alice Rostom de Mello

**Affiliations:** 1 Institute of Biosciences, Department of Physical Education, São Paulo State University, Unesp, SP, Brazil; 2 Institute of Biosciences, Department of Physiology and Biophysical, Campinas State University, Unicamp, SP, Brazil

## Abstract

**Background:**

Ninety percent of cases of diabetes are of the slowly evolving non-insulin-dependent type, or Type 2 diabetes. Lack of exercise is regarded as one of the main causes of this disorder. In this study we analyzed the effects of physical exercise on glucose homeostasis in adult rats with type 2 diabetes induced by a neonatal injection of alloxan.

**Methods:**

Female Wistar rats aged 6 days were injected with either 250 mg/kg of body weight of alloxan or citrate buffer 0.01 M (controls). After weaning, half of the animals in each group were subjected to physical training adjusted to meet the aerobic-anaerobic metabolic transition by swimming 1 h/day for 5 days a week with weight overloads. The necessary overload used was set and periodically readjusted for each rat through effort tests based on the maximal lactate steady state procedure. When aged 28, 60, 90, and 120 days, the rats underwent glucose tolerance tests (GTT) and their peripheral insulin sensitivity was evaluated using the HOMA index.

**Results:**

The area under the serum glucose curve obtained through GTT was always higher in alloxan-treated animals than in controls. A decrease in this area was observed in trained alloxan-treated rats at 90 and 120 days old compared with non-trained animals. At 90 days old the trained controls showed lower HOMA indices than the non-trained controls.

**Conclusion:**

Neonatal administration of alloxan induced a persistent glucose intolerance in all injected rats, which was successfully counteracted by physical training in the aerobic/anaerobic metabolic transition.

## Background

Diabetes mellitus is caused by the reduced secretion and/or diminished action of insulin. Diabetic individuals fall into two distinct groups, those in which the disease is caused by a lack of insulin – known as insulin-dependent diabetes mellitus or IDDM – or by a certain resistance to the action of insulin, known as non-insulin-dependent diabetes mellitus or NIDDM.

NIDDM develops in a slow and delayed manner, and accounts for about 90% of the cases of diabetes. The main causes of type 2 diabetes include obesity, high calorie intake and a sedentary lifestyle [1].

Insulin resistance inhibits glucose consumption by muscles and adipose tissues, and stimulates glucose release by the liver, leading to hyperglycemia. There is evidence that insulin resistance may be the first derangement in cases of diabetes, preceding the onset of hyperglycemia [2].

In 1989, Portha *et al*. developed an experimental model for testing insulin resistance by administering streptozotocin to newly born rats. This caused transitory hyperglycemia in the rats, with basal serum glucose levels returning to normal values in the first week of their lives, after the production of insulin by β cells normalized [3]. Later, Kodama *et al*. [4] described another mode of inducing insulin resistance in rats by substituting streptozotocin for alloxan [4]. In this model, alloxan was administered to Wistar rats of 2, 4, or 6 days of age. When they reached the age of 60 days, all rats that had received alloxan on their 2^nd ^day of life showed slightly increased non-fasting serum glucose, whereas those that had received the drug on the 4^th ^or 6^th ^day of life showed significantly higher plasma glucose levels than did the controls.

Unlike the rats treated with streptozotocin, those treated with alloxan on the 4^th ^or 6^th ^day of life showed increased longevity coupled with persistent hyperglycemia, since insulin production by pancreatic β cells did not normalize.

In a previous study we analyzed the model developed by Kodama *et al*. [4] and observed that all male Wistar rats which had received alloxan (200 mg/kg body weight) at 2 days old managed to recover from the glucose intolerance on the 90^th ^day [5], while other metabolic disorders, such as high levels of circulating Free Fatty Acid (FFA), persisted during the experimental period.

Regular physical activity has been proved to be efficient in normalizing glucose homeostasis in diabetic patients, as well as improving insulin sensitivity and glucose tolerance [6-8]. Aerobic exercises are generally prescribed to patients with type 2 diabetes [6,7,9]. Since there are limitations to experimentation with humans, the use of animal models is necessary when testing these strategies.

The aim of the present study was to analyze the effects of a physical exercise programme, adjusted to the aerobic-anaerobic transition, on glucose homeostasis in adult Wistar rats treated with alloxan when newly born.

## Methods

### Animal treatment

The studies were carried out using newly born female Wistar rats, kept at 25 ± 1°C and under a 12/12 h light-dark cycle. The animals were fed on a special purified diet for rodents prepared in our laboratory (following AIN-93G [10]) and they were allowed water *ad libitum*. Food and water intake as well as the body weight of all animals were recorded weekly. All animal procedures complied with the European Convention for the Protection of Vertebrate Animals used for Experimental and other Scientific Purposes of the Council of Europe, n 123, Strasbourg, 1985.

### Administration of alloxan

On reaching the age of 6 days, 16 female rats were injected intraperitoneally with alloxan monohydrate (Sigma-Aldrich Inc., St Louis, MO, USA) dissolved in citrate buffer 0.01 M, pH 4.5 to a concentration of 250 mg/kg of body weight. The animals were fasted for 16 h prior to drug administration [4]. Sixteen rats of the same age injected with the same volume of citrate buffer were used as controls. All the experimental procedures were carried out as originally proposed by Kodama *et al *[4], except that those authors injected a lower dose of alloxan (200 mg/kg of body weight). Previous studies in our laboratory [5] showed that using alloxan 200 mg/kg of body weight resulted in spontaneous recovery from glucose intolerance in all rats after 90 days of experimentation.

### Experimental groups

The animals (both controls and alloxan-treated) were randomly divided into groups of 8 after weaning (28 days), and were treated up to 120 days old as follows:

• *Controls (C*): no exercise programme;

• *Trained Controls (TC): *underwent a swimming training programme;

• *Alloxan (A*): no exercise programme;

• *Trained Alloxan (TA): *underwent a swimming training programme;

### Glucose tolerance test (GTT)

All rats were fasted for 16 h when they reached the age of 28, 60, 90, and 120 days, then a glucose solution (200 g/L) was administered via a catheter through the mouth into the stomach at a dose of 2.0 g/kg of body weight. Blood samples (25 μL) were then taken from a cut on the tip of their tails to determine the levels of serum glucose at 0, 30, 60, and 120 min after glucose administration. In addition, blood samples (75 μL) were obtained at 0 min to obtain insulin levels in order to calculate the HOMA (Homeostasis Model Assessment) insulin sensitivity index.

The levels of serum glucose were determined using the glucose-oxidase colorimetric enzymatic method (Laborlab Kit, Guarulhos, São Paulo, Brazil), while levels of insulin were measured by radioimmunoassay [11]. The blood response to glucose during the GTT was evaluated based on the total area under the curve of serum glucose (mM × 120 min) [12].

### Sensitivity to insulin

Peripheral insulin sensitivity was evaluated on days 28, 60, 90, and 120 based on the HOMA insulin sensitivity index using the following equation: serum insulin (pmol/L) × serum glucose mmol/L/22.5. Using this method, higher HOMA indices denoted lower insulin sensitivity [13].

### Effort test

On the 28th day, all animals were subjected to an effort trial to determine their individual exercise intensity necessary to reach the aerobic/anaerobic metabolic transition, following the Maximal Lactate Steady State protocol (MLSS). This protocol was designed to detect the highest blood lactate concentration at which the entrance of lactate into the blood stream was counterbalanced by its removal, maintaining a stable concentration during exercises of constant intensity [14]. This has proved useful in prescribing exercises to and determining the aerobic capacity of humans [15], rats [16] and mice [17]. Our research group has recently designed a MLSS protocol for rats using swimming exercises [18], which was employed in this study. In short, to determine the MLSS, a series of 25 min swimming exercises were performed, which supported increasing overloads in relation to body weight, and were fixed in each series, with intervals of 48 h between them. Blood samples were taken every 5 min, from a cut on the tail tip, for lactate determination. The blood lactate concentration representative of the MLSS was considered at the highest overload in which there was no variation in the blood lactate higher than 1.0 mmol/L between 10 and 25 min of exercise [18,19]

### Training Programmes

The animals were then subjected to a training programme of 60 min/day, 5 days a week, over 12 weeks. The swimming sessions were run in individual tanks with the established relative overloads according to the rat's weight in order to reach the individual aerobic/anaerobic metabolic transition [18]. Every 30 days, new MLSS tests were conducted to assign new overloads.

### Statistics

Results are presented as means ± standard deviation and were analyzed using the Students t-test and two-way ANOVA followed by a Newman-Keuls test, where appropriate. Significance level was 5%.

## Results

### General parameters

Body weight, food and water intake are illustrated in Table 1. Groups TC [F_1,24 _= 9.33], A [F_1,24 _= 11.0] and TA [F_1,24 _= 11.35] displayed a smaller area under the body weight curve than the controls. Rats in the TA [F_1,18 _= 16.66] group consumed more food than the sedentary rats in groups C and A. No difference in water intake was found between the groups.

**Table 1:** Body weight, food intake and water intake.

**Groups**	**Body Weight**	**Food intake**	**Water intake**
C	3648.7 ± 212.4	103.0 ± 11.0	127.9 ± 21.2
TC	3281.2 ± 212.0^a^	131.3 ± 8.7	148.6 ± 22.6
A	3258.3 ± 202.2^b^	106.9 ± 6.5	124.4 ± 5.2
TA	3093.2 ± 291.2^c^	137.6 ± 19.6^c,d^	156.9 ± 4.1

Areas under the body weight (grams × 12 weeks), food intake (food grams/100 g of body weight × 12 weeks) and water intake (water ml/100 g of body weight × 12 weeks) curves of the animals.

Values are mean ± standard deviation, n = 8 animals per group.

C = Control; TC = Trained control; A = Alloxan e TA = Trained alloxan a = C vs TC; b = C vs A; c = C vs TA; d = A vs TA

### Prior-to-training evaluations

**Figure 1:**
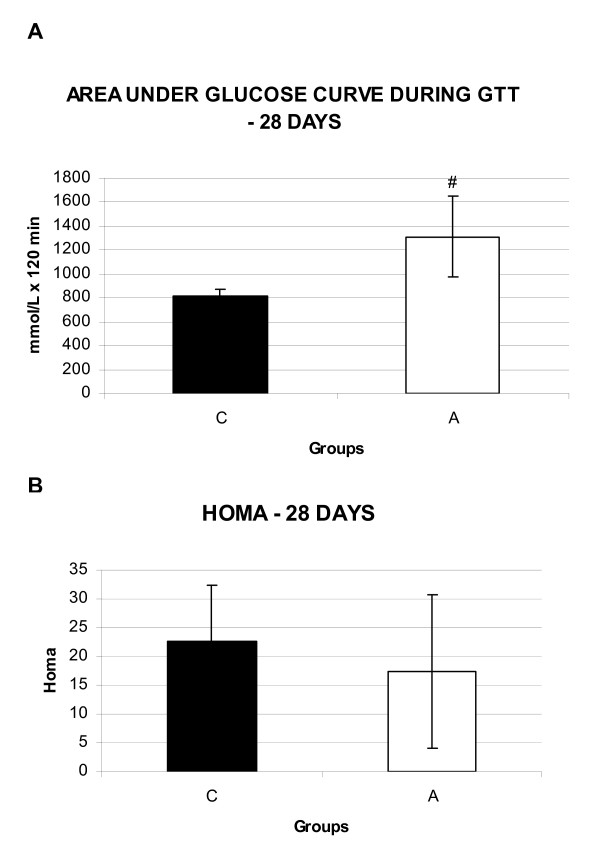
**Area under serum glucose curve during GTT and HOMA – 28 days**. **A**- Area under serum glucose curve during GTT (mmol/L × 120 min) of the animals after weaning (28 days of age – C and A). **B**- HOMA* insulin sensitivity index after weaning (28 days of age – C and A). Values showed as mean ± standard deviation, n = 8 animals per group. C = Control; A = Alloxan. *homeostasis assessment model. # diference.

The results of the GTT on the 28th day are presented on Figure 1A. Blood serum glucose levels in rats that received alloxan were higher (A = 1309.0 ± 337.8, p < 0.05, student t-test) than those of controls (C = 817.6 ± 59.6, p < 0.05 student t-test), as reflected by the increase in the area under the serum glucose curves (mmol/L × 120 min) in this group. There was no difference between the HOMA insulin sensitivity indices of both groups (C = 22.7 ± 9.7 and A = 17.4 ± 13.2, p < 0.05, student t-test) when evaluated on the 28th day (Figure 1B).

**Figure 2:**
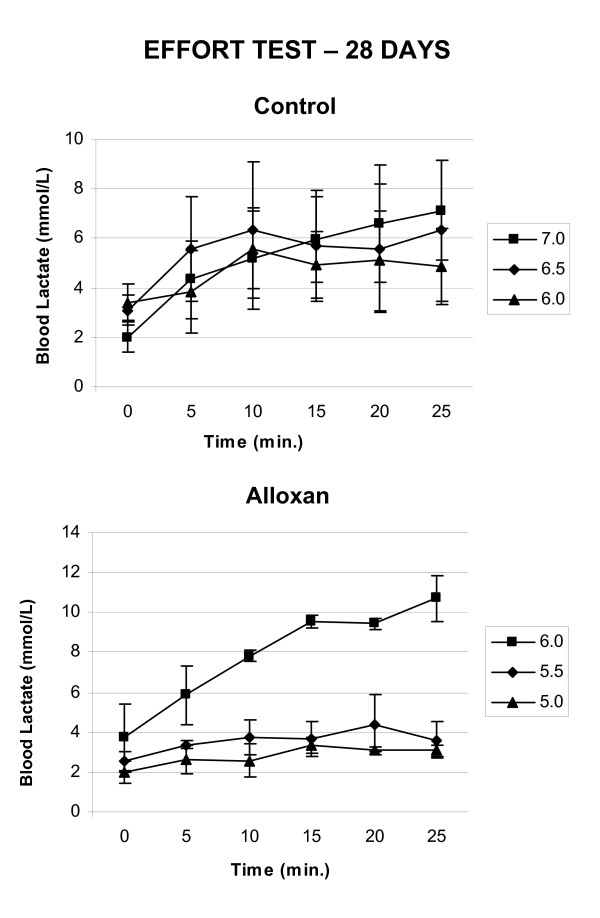
**Effort test**. Blood lactate concentrations (mmol/L) during the effort tests for determining MLSS of most 28-days-old rats of each group.

On the 28th day, all rats were subjected to the described MLSS protocol. MLSS was performed using overloads of 5.0–7.5% of the rats' body weights. The animals did not reach the MLSS using the same overloads within the same group. Figure 2 represents the lactate concentration kinetics during exercise for most animals in each group. In the TC group, 25% of the animals reached the MLSS using overloads of about 5% of their body weight, and their mean lactate concentration was 7.3 ± 2.4 mmol/L; the remaining 75% (Figure 2) reached the MLSS using overloads of 6.5% of their body weight and their mean lactate concentration was 6.0 ± 2.6 mmol/L. In the TA group, 50% of the animals (Figure 2) reached the MLSS using 5.5% of body weight overloads and their mean lactate concentration was 3.8 ± 0.7 mmol/L, while 33.3% of the animals reached the MLSS with 6.0% of body weight overloads and their mean lactate concentration was 6.5 ± 0.6 mmol/L, and 16.7% of the animals reached the MLSS using loads 6.5% of their body weight, with a mean lactate concentration of 10.4 ± 0.6 mmol/L.

### During training evaluations

There were no differences between the HOMA indices of the groups at 60 days old (C = 17.7 ± 3.6, TC = 19.8 ± 4.7; A = 33.3 ± 19.6 and TA = 32.4 ± 10.6). However, on the 90^th ^day, TC animals were more insulin sensitive than the other groups (TC = 19.8 ± 2.6 [F_1,19 _= 6.96]; C = 28.7 ± 4.5; A = 30.8 ± 4.8 and TA = 31.2 ± 5.5), and on the 120^th ^day, no statistical differences between the groups regarding insulin sensitivity were observed (C = 19.4 ± 4.3; TC = 17.4 ± 6.4; A = 19.4 ± 6.4 and TA = 18.6 ± 2.6).

**Figure 3:**
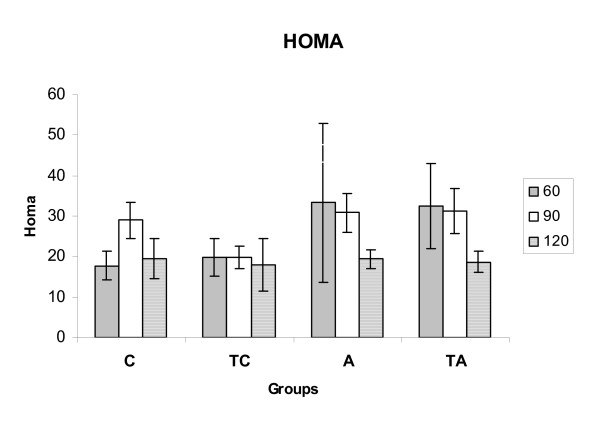
**HOMA**. HOMA* insulin sensitivity index of 60th, 90th and 120th days of age (C, TC, A and TA). Values showed as mean ± standard deviation, n = 8 animals per group. C = Control; TC = Trained control; A = Alloxan e TA = Trained alloxan. a = C vs TC; e = TC vs TA; *homeostasis assessment model.

**Figure 4:**
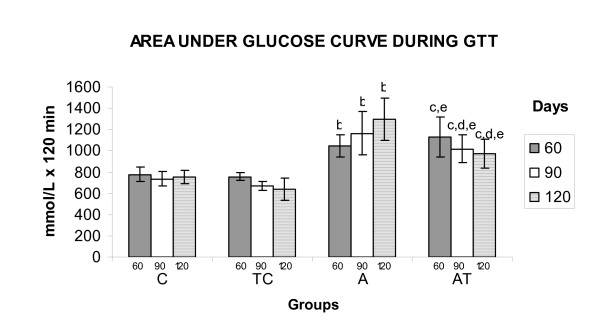
**Area under serum glucose curve during GTT**. Area under serum glucose curves during GTT (mmol/L × 120 min) of 60th, 90th and 120th days of age (C, TC, A and TA). Values showed as mean ± standard deviation, n = 8 animals per group. C = Control; TC = Trained control; A = Alloxan e TA = Trained alloxan. b = C vs A; c = C vs TA; d = A vs TA; e = TC vs TA.

At 60 days old, groups A (1043.4 ± 103.1 [F_1,26 _= 61.97) and TA (1131.2 ± 189.7 [F_1,26 _= 61.97]) showed high serum glucose concentrations based on the area under the serum glucose curve during the GTT, compared with the controls (C = 774.9 ± 69.9 and TC = 756.9 ± 38.9). On the other hand, at 90 days old (C = 735.9 ± 64.1; TC = 670.9 ± 40.8; A = 1162.9 ± 202.9 and TA = 1017.4 ± 129.0 [F_1,26 _= 5.55]) and 120 days old (C = 755.8 ± 64.5; TC = 640.7 ± 106.4; A = 1299.4 ± 198.3 and TA 972.2 ± 135.0) the area under the serum glucose curve in the TA [F_1,26 _= 4.43] group decreased compared with that in group A (Figure 4).

## Discussion

The aim of the present study was to analyze the effects of a physical exercise programme, adjusted to the aerobic-anaerobic transition, on the glucose homeostasis of adult Wistar rats treated with alloxan when newly born.

Our results demonstrated that the sedentary group C showed a greater increase in body weight than did groups A, TA, and TC. Although TA rats had the highest food intake, they did not assimilate this energy as body mass.

It was previously shown that animals receiving streptozotocin in their first days of life had compromised secretory insulin capacity just after drug administration [20]. It is probable that a similar reduction in insulin production took place in this study when the rats were injected with alloxan, because animals in this group demonstrated a low increase in body weight. Reports from other studies have shown that young animals or humans with reduced insulin demonstrated body weight gain impairment and a delay in the process of growth and physical maturation [21].

Obesity is one of the main causes of type 2 diabetes. In the present study, the training programme reduced body weight gain. These results are in agreement with other studies [22] which showed that animals subjected to running sessions gained 24% less body weight than sedentary controls following 6 weeks of physical training. The literature also states that physical activity on a regular basis and restrictions in body weight gain reduce the incidence of type 2 diabetes in patients with glucose intolerance [23].

The GTT on the 28th day illustrated that the animals exhibited an adequate response to neonatal alloxan administration, via a bigger area under the serum glucose curve than the controls. In the subsequent evaluations at 60, 90 and 120 days old, group A rats remained glucose intolerant.

In another study carried out in our laboratory, Oliveira *et al *(2005) observed that rats receiving alloxan (200 mg/kg of body weight) at 2 days old showed spontaneous improvement in glucose tolerance [5]. In the present study, the observed high steady blood serum glucose levels in group A animals was probably due to the later age at which the animals received alloxan (6 days) and also because of the higher dosage used (250 mg/kg). Kodama *et al*. (1993) showed that when alloxan was administered to newly born rats, the earlier the drug was administered the less severe the blood glucose derangement was in later life [4].

According to the American Diabetes Association, patients who present with glucose intolerance and/or altered fasting blood glucose levels are prone to developing type 2 diabetes, and are thus candidates for interventions in order to monitor and control the onset of the disease [24]. The American Diabetes Association also claims that oral glucose tolerance tests and evaluation of fasting blood glucose levels are the best parameters to track the development of type 2 diabetes.

The United Kingdom Prospective Diabetes Study (1995) demonstrated that β cell dysfunction in humans starts at around 10–12 years old before type 2 diabetes develops, therefore offering a long interval for the initiation of prophylactic interventions [25]. The non-exercised alloxan rats in the present study remained in this pre-diabetic condition throughout the experiment, illustrating the usefulness of this model in designing intervention strategies in cases of type 2 diabetes in future studies, since there are obstacles to experimentation with humans.

Identification of the MLSS was successfully employed to evaluate the aerobic capacity of the experimental animals [18]. This protocol also enabled the quantification of their individual aerobic capacity. In the present study, the procedures employed to induce glucose intolerance caused a reduction in blood lactate levels in most TA animals at the MLSS compared with controls, even though the overloads during the MLSS were the same for both groups.

Randle, in studies using the hearts and diaphragms of rats suggested the existence of a mechanism known as the glucose-fatty acid cycle. He observed that increases in FFA plasma levels were followed by decreases in glucose uptake and oxidation by these organs [26]. In general, type 2 diabetes leads to an increase in the circulating levels of FFA, which are used up by peripheral tissues, such as the skeletal muscles, saving glycogen [27]. This may have led to reduced glucose utilization, thus resulting in a lower production of lactate in TA rats.

Our results show that when the animals were 90 days old, i.e. 60 days after the start of physical training, the swimming sessions effectively reduced the area under the serum glucose curve of the GTT in TA rats, thus demonstrating a significant recovery in tolerance to glucose (Figure 4). A similar recovery was observed in TA rats at 120 days old. The reduction in serum glucose levels in the TA group could have been mediated by an increase in the numbers of glucose transporters [28]. A previous study conducted in our laboratory demonstrated that swimming sessions in the aerobic/anaerobic transition also attenuated glucose intolerance in type 1 diabetes rat models [29].

Knowing the exact moment and how physical activity can contribute to counteracting or preventing type 2 diabetes will be useful in encouraging diabetic patients to take part in physical activity on a regular basis. Page *et al*. (1992) showed that 6 months of continued regular exercise was not enough time for patients to assimilate this into their life habits [30].

## Conclusion

The neonatal administration of alloxan induced persistent glucose intolerance, which was counteracted by physical training in the aerobic/anaerobic metabolic transition. The use of the HOMA index as an indicator of insulin resistance proved inefficient in detecting differences among the experimental groups in most cases. Thus complementary studies using alternative procedures are necessary in order to obtain a more comprehensive characterization of this model.

## Abbreviations

GTT: Glucose Tolerance Test; MLSS: Maximal Lactate Steady State; HOMA: homeostasis model assessment; FFA: Free fatty acid; IDDM: insulin-dependent diabetes mellitus; NIDDM: non-insulin-dependent diabetes mellitus.

## Competing interests

The authors declare that they have no competing interests.

## Authors' contributions

CSAM, conceived of the study, developed the study protocol, reviewed the references, abstracted data, analyzed the data, and wrote the paper. CR, GGA, MBA, FBM-G, CAMO, FAV and EL, participated in the design of the study, reviewed the manuscript, collaborated in the biochemical dosages, and advised on revisions to the manuscript. MARM. conceived of the study, gave financial support and participated in its design and coordination and helped to draft the manuscript. All authors read and approved the final manuscript.

## Pre-publication history

The pre-publication history for this paper can be accessed here:


